# Post-weaning diet determines metabolic risk in mice exposed to overnutrition in early life

**DOI:** 10.1186/1477-7827-12-73

**Published:** 2014-08-01

**Authors:** Vicky King, Jane E Norman, Jonathan R Seckl, Amanda J Drake

**Affiliations:** 1 MRC/University of Edinburgh Centre for Reproductive Health, Queen’s Medical Research Institute, 47 Little France Crescent, Edinburgh EH16 4TJ, UK; 2 Endocrinology Unit, University/BHF Centre for Cardiovascular Science, University of Edinburgh, QMRI, 47 Little France Crescent, Edinburgh EH16 4TJ, UK

**Keywords:** Maternal overnutrition, Obesity, Developmental programming

## Abstract

**Background:**

Maternal overnutrition during pregnancy is associated with an increased risk of obesity and cardiometabolic disease in the offspring; a phenomenon attributed to ‘developmental programming’. The post-weaning development of obesity may associate with exacerbation of the programmed metabolic phenotype. In mice, we have previously shown that exposure to maternal overnutrition causes increased weight gain in offspring before weaning, but exerts no persistent effects on weight or glucose tolerance in adulthood. In order to determine whether post-weaning exposure to a cafeteria diet might lead to an exacerbation of programmed effects, offspring born and raised by mothers on control (CON) or cafeteria (DIO) diets were transferred onto either CON or DIO diets at weaning.

**Findings:**

Post-weaning DIO caused the development of obesity, with hyperglycaemia and hyperinsulinaemia in males; and obesity with hyperinsulinaemia in females and with increased cholesterol levels in both sexes. Exposure to maternal overnutrition during pregnancy and lactation caused only subtle additional effects on offspring phenotype.

**Conclusions:**

These results suggest that post-weaning exposure to a high-fat high-sugar diet has a more profound effect on offspring weight gain and glucose tolerance than exposure to maternal overnutrition. These data emphasise the importance of optimising early life nutrition in offspring of both obese and lean mothers.

## Findings

### Background

Human and animal studies have shown that the environment in early life can increase the risk of later metabolic disease [1]. There is increasing interest in the role of maternal obesity in the ‘programming’ of offspring disease risk [2] and recent studies have shown that maternal obesity and gestational weight gain are independently associated with offspring cardiometabolic risk and with all-cause mortality [3,4]. This is of substantial importance given the increasing prevalence of obesity worldwide, including amongst women of childbearing age [5]. In order to understand the mechanisms by which exposure to maternal obesity leads to programming of offspring phenotype, animal models have been developed, many of which recapitulate the findings in human studies, showing effects on offspring adiposity, glucose-insulin homeostasis, blood pressure and appetite [6-8].

Using a mouse model, we recently reported remarkably few effects of maternal overnutrition on body weight and metabolism in the directly exposed (F1) offspring [9]. Despite this, there were effects on birthweight and metabolism in a second generation, suggesting that there were persistent effects in F1 offspring leading to the transmission of effects [9]. Since in humans, postnatal obesity appears to be an important determinant of metabolic disease [10], and post-weaning exposure to a high-fat diet is associated with amplification of effects in some animal models [8], we hypothesised that *post-weaning* exposure to a cafeteria diet would result in amplification of the phenotype in both male and female F1 offspring of overnourished mothers.

## Methods

Animal studies were conducted as previously reported [9] under approval by the UK Home Office, under the Animals (Scientific Procedures) Act. The experiments were set up as previously described, using a new cohort of mice. From 5 weeks, female C57BL/6 mice were allowed free access to cafeteria (DIO: 58 kcal% fat, 25.5 kcal% carbohydrate as sucrose) or matched control diets (Con: 10.5 kcal% fat and 73.1 kcal% carbohydrate as corn-starch) (Diets D12331 and D12328, Research Diets, New Brunswick, USA). At 17 weeks, females were time-mated with chow-fed C57BL/6 males (RMI 801002, Special Diets Services, Witham, UK). Females remained on experimental diets through pregnancy and lactation. At postnatal day 1, litters were weighed and reduced to five pups; animals remained with their biological mothers until weaning at 3 weeks. Groups of F1 male and female pups were selected randomly from each litter and weaned onto cafeteria (D12331) or control diets (D12328). This gave four groups of F1 offspring (n = 7-8/group): 1) offspring of Con mothers weaned onto control diet (CON/CON) 2) offspring of Con mothers weaned onto cafeteria diet (CON/DIO) 3) offspring of DIO mothers weaned onto control diet (DIO/CON) and 4) offspring of DIO mothers weaned onto cafeteria diet (DIO/DIO).

Intraperitoneal glucose tolerance testing (GTT) and lipid measurements were performed at 3 and 6 months following a 6-hour fast. A fasting tail blood sample was taken immediately prior to glucose injection after which mice received an intraperitoneal injection of glucose (2 g/kg body weight). Tail blood samples were collected at 15, 30, 60 and 90 minutes, placed on ice, centrifuged at 2.3 × g for 10 minutes at 4°C and the supernatant plasma stored at -20°C. Plasma glucose levels were determined by the hexokinase/glucose-6-phosphate dehydrogenase method (Thermo Fisher Scientific, UK) and plasma insulin by ELISA (Crystal Chem Inc., Downers Grove, IL, USA). We calculated homoeostasis model assessment of insulin resistance (HOMA-IR; fasting plasma glucose [mmol/L] × fasting insulin [mU/L])/22·5). Fasting plasma cholesterol and triglyceride levels were measured by an enzymatic assay following the manufacturer’s instructions (Infinity kits; Thermo Fisher Scientific, UK).

Data are expressed as mean ± SEM. Groups were compared by independent t-tests, Area under Curve, repeated measures ANOVA and two-way ANOVA as appropriate. Data for plasma parameters and organ weights were compared by with pre-weaning and post-weaning diet as the main factors using Statistica (Statsoft) or Graphpad prism version 5.

## Results

Females weaned onto cafeteria diets were heavier than controls at mating (Con 21.5 ± 0.3 g; DIO 28.0 ± 1.4 g; p < 0.001) in agreement with our previous study [9]. There were no differences in gestation length (Con 20.2 ± 0.2; DIO 20.4 ± 0.2 days; p = 0.5) or litter numbers between groups or in birthweight in either sex (Table 1). By weaning, both male and female offspring of DIO were heavier than offspring of Con mothers (Table 1). At 3 and 6 months, male and female CON/DIO and DIO/DIO offspring were heavier than either CON/CON or DIO/CON (repeated measures ANOVA; effect of post-weaning diet: males F(1,28) = 91.3, p < 0.001, females F(1,27) = 97.3, p < 0.001) but there was no additional effect of exposure to maternal overnutrition during pregnancy and lactation. At 6 months, post-weaning exposure to cafeteria diet was associated with increased fat pad weight in both males and females (Table 1) and there was an additional effect of exposure to maternal overnutrition during pregnancy and lactation to increase retroperitoneal fat pad weight (F(1,27) = 5.79, p = 0.02) specifically in female offspring (Table 1).

**Table 1:** Body weight, plasma parameters and organ weights in offspring

	**CON male (n)**		**DIO male (n)**		**p value**	**CON female (n)**		**DIO female (n)**		**p value**
	**n = 22 from 9 litters**		**n = 16 from 10 litters**			**n = 18 from 9 litters**		**n = 19 from 10 litters**		
Birthwt (g) (litter mean)	1.37 +/- 0.04		1.30 +/- 0.11		0.54	1.30 +/- 0.06		1.21 +/- 0.07		0.39
Wean wt (g) (litter mean)	8.42 +/- 0.53		10.54 +/- 0.28		0.002	7.91 +/- 0.20		9.70 +/- 0.20		<0.001
**Post-wean group**	CON/CON	CON/DIO	DIO/CON	DIO/DIO	p value	CON/CON	CON/DIO	DIO/CON	DIO/DIO	p value
	male n = 8	male n = 8	male n = 8	male n = 8		female n = 8	female n = 8	female n = 8	female n = 7	
**3 months**										
Plasma cholesterol mmol/l	2.47 +/- 0.34	5.28 +/- 0.57	2.73 +/- 0.20	7.01 +/- 0.39	a:p < 0.001	3.16 +/- 0.50	4.67 +/- 0.53	2.47 +/- 0.23	3.89 +/- 0.34	a:p = 0.003
										b:p = ns
					b:p = 0.016					
Plasma TG mmol/l	0.57 +/- 0.06	0.66 +/- 0.10	0.53 +/- 0.11	0.63 +/- 0.83	a:p = ns	0.61 +/- 0.06	0.72 +/- 0.08	0.56 +/- 0.07	0.52 +/- 0.11	a:p = ns
										b:p = ns
					b:p = ns					
Plasma glucose AUC	985 +/- 55	1668 +/- 203	1056 +/- 108	2037+/- 139	a:p < 0.001	898 +/- 109	1087 +/- 123	1026 +/- 121	1197 +/- 97	a:p = ns
										b:p = ns
					b:p = ns					
Plasma insulin AUC	74 +/- 6	247 +/- 22	77 +/- 11	177 +/- 16	a:p < 0.001	60 +/- 5	110 +/- 13	57 +/- 3	90 +/- 12	a:p < 0.001
										b:p = ns
					b:p = 0.03					
HOMA IR	1.55 +/- 0.12	5.43 +/- 0.57	1.61 +/- 0.24	4.19 +/- 0.58	a:p < 0.001	1.27 +/- 0.07	2.43 +/- 0.33	1.16 +/- 0.06	1.65 +/- 0.22	a:p00.01
										b:p = 0.044
					b:p = ns					
**6 months**										
Plasma cholesterol mmol/l	2.87 +/- 0.39	9.37 +/- 0.46	4.06 +/- 0.29	7.77 +/- 0.51	a:p < 0.001	3.92 +/- 0.38	6.65 +/- 0.55	2.98 +/- 0.41	6.26 +/- 0.26	a:p < 0.001
										b:p = ns
					b:p = ns					
Plasma TG mmol/l	0.55 +/- 0.09	0.71 +/- 0.05	0.63 +/- 0.06	0.71 +/- 0.07	a:p = ns	0.63 +/- 0.08	0.83 +/- 0.03	0.51 +/- 0.10	0.66 +/- 0.09	a:p = 0.045
										b:p = ns
					b:p = ns					
Plasma glucose AUC	1189 +/- 125	2287 +/- 280	1205 +/- 163	2074 +/- 143	a:p < 0.001	1005 +/- 92	1420 +/- 163	886 +/- 1487	1487 +/- 162	a:p < 0.001
										b:p = ns
					b:p = ns					
Plasma insulin AUC	109 +/- 17	581 +/- 61	129 +/- 14	535+/-52	a:p < 0.001	78 +/- 13	169 +/- 29	66 +/- 6	173 +/- 16	a:p < 0.001
										b:p = ns
					b:p = ns					
HOMA-IR	2.24 +/- 0.34	11.56 +/- 1.17	2.77 +/- 0.32	10.47 +/- 0.92	a:p < 0.001	1.57 +/- 0.31	3.41 +/- 0.58	1.38 +/- 0.13	3.72 +/- 0.38	a:p < 0.001
										b:p = ns
					b:p = ns					
Liver	4.11 +/- 0.10	3.70 +/- 0.52	3.98 +/- 0.10	4.24 +/- 0.38	a:p = ns	4.02 +/- 0.19	3.68 +/- 0.10	4.04 +/- 0.09	3.63 +/- 0.16	a:p = 0.014
										b:p = ns
					b:p = ns					
RP fat pad	0.29 +/- 0.08	0.97 +/- 0.07	0.30 +/- 0.02	0.94 +/- 0.10	a:p < 0.001	0.28 +/- 0.05	0.97 +/- 0.18	0.31 +/- 0.07	1.56 +/- 0.16	a:p < 0.001
										b:p = 0.023
					b:p = ns					
Mes fat pad	0.42 +/- 0.09	1.39 +/- 0.14	0.74 +/- 0.07	1.40 +/- 0.24	a:p < 0.001	0.37 +/- 0.06	0.76 +/- 0.10	0.45 +/- 0.10	1.02 +/- 0.10	a:p < 0.001
										b:p = ns
					b:p = ns					
SC fat pad	0.96 +/- 0.11	2.92 +/- 0.20	0.92 +/- 0.06	2.74 +/- 0.24	a:p < 0.001	0.75 +/- 0.09	2.44 +/- 0.23	0.80 +/- 0.12	2.44 +/- 0.11	a:p < 0.001
										b:p = ns
					b:p = ns					
Epi fat pad	0.90 +/- 0.13	2.26 +/- 0.14	0.87 +/- 0.14	2.28 +/- 0.15	a:p < 0.001					
					b:p = ns					

Organ weights expressed as a% of body weight (TG = triglycerides, RP = retroperitoneal fat, Mes = mesenteric fat, SC = subcutaneous fat, Epi = epididymal fat). a = effect of postnatal diet, b = effect of maternal obesity for all groups compared to CON/CON.

**Figure 1:**
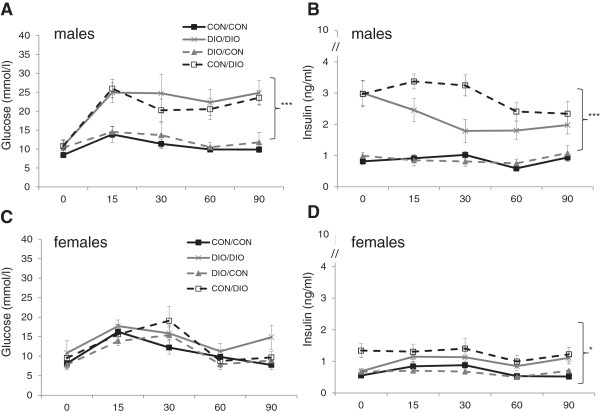
**Glucose tolerance tests in offspring at 3 months of age.** Plasma glucose and insulin concentrations in males **(Figure**1**A and B)** and females **(Figure**1**C and D)**. Data are mean ± SEM and were analysed by ANOVA analysis of Area Under Curve, (n = 7-8/group) *p < 0.05, ***p < 0.01 for CON/DIO and DIO/DIO vs CON/CON.

At 3 months, post-weaning exposure to cafeteria diet increased glucose, insulin (Figure 1A and B) and cholesterol (Table 1) concentrations in males; increased plasma insulin and cholesterol concentrations in females (Figure 1C and D) and increased HOMA-IR in both sexes (Table 1: males: F(1,25) = 60.9, p < 0.001; females F(1,25) = 15.1, p < 0.001). There was an additional effect of exposure to maternal overnutrition to reduce insulin levels in males (Figure 1B. F(1,28) = 5.2, p = 0.03) and to reduce HOMA-IR in females (Table 1: F(1,25) = 4.48, p = 0.044).

**Figure 2:**
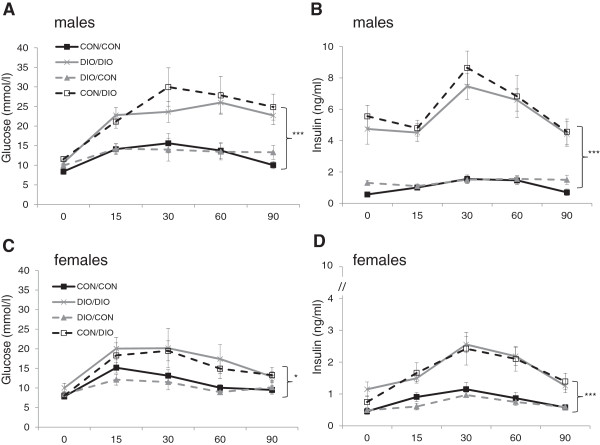
**Glucose tolerance tests in offspring at 6 months of age.** Plasma glucose and insulin concentrations in males **(Figure**2**A and B)** and females **(Figure**2**C and D)**. Data are mean ± SEM and were analysed by ANOVA analysis of Area Under Curve, (n = 7-8/group), *p < 0.05, ***p < 0.01 for CON/DIO and DIO/DIO vs CON/CON.

At 6 months, post-weaning exposure to cafeteria diet increased glucose, insulin (Figure 2) and cholesterol (Table 1) concentrations and HOMA-IR in both sexes (Table 1: males: F(1,28) = 118.7, p < 0.001; females F(1,26) = 28.7, p < 0.001). There were no persistent effects of exposure to maternal overnutrition during pregnancy and lactation on plasma glucose, insulin or cholesterol levels or HOMA-IR in either sex.

## Discussion

Here we replicate the findings of our earlier study in which we found that maternal overnutrition was associated with few effects in the F1 offspring [9]. Nevertheless, since some studies report that the programming phenotype may only become overt, or is amplified following postnatal exposure to a high-fat diet [11,12]; we reasoned that post-weaning exposure to a cafeteria diet might result in a more obvious phenotype. As expected, F1 males and females weaned onto the cafeteria diet showed increased weight gain, glucose-insulin dyshomeostasis and hypercholesterolaemia. There were some additional effects of exposure to maternal overnutrition at 3 months which were sex-specific, with reduced insulin levels in males and reduced HOMA-IR in females, suggesting increased insulin sensitivity. However, these effects were no longer present at 6 months, although maternal overnutrition was associated with increased retroperitoneal fat mass in F1 females on the cafeteria diet.

Our results differ from those in number of animal models which document effects of exposure to a maternal high-fat diet on offspring obesity and glucose-insulin homeostasis [7,13,14]. Nevertheless, our findings are consistent with reports in some models which show little additional effect of maternal overnutrition on the phenotype induced by post-weaning exposure to an obesogenic diet [15-18]. Notably, in one of these studies, maternal consumption of a high-fat diet was *protective* against the development of obesity induced by a sucrose-rich diet [17,18]. Potential mechanisms for the apparent ‘protection’ from the adverse consequences of early life exposure to overnutrition may include differences in the species or strain of animal used, the degree of maternal obesity and/or gestational weight gain, age at mating and whether first or second litters are used. The diets utilised in different studies differ in macro- and micronutrient content and this may be of particular importance in determining effects on the offspring [17,19,20]. We used diets matched for micronutrient content, which may represent an additional source of difference between groups in other studies. For example, Couvreur et al. showed that the offspring of females fed a high-fat diet were protected from the development of obesity postnatally, whereas the offspring of mothers maintained on a highly palatable diet were not [17]; whilst another group reported that different fat sources in the maternal diet had very different influences on the susceptibility of offspring to body weight gain, with prenatal exposure to some fat types providing relative protection from the development of obesity [21]. We have previously documented in our model that maternal protein intake does not differ between groups [9], however it is possible that in other studies, the dietary protein content differs between obesogenic and control diets, so that plausibly, some of the programmed effects might instead be due to *in utero* protein restriction which has known programming effects [22]. Additionally, unlike other studies which use an obesogenic diet alongside standard laboratory chow, or which supplement diets with highly palatable substances to increase calorie availability [7,23], we used diets matched for micronutrient content, which may represent an additional source of difference between groups in other studies. A further possibility is that there may a ‘ceiling effect’ from such a high-fat diet (58% fat), and that more subtle effects may be seen with more moderate diets.

Another explanation for the lack of phenotype in our model could be that the moderate degree of maternal obesity engendered by the cafeteria diet was not severe enough to affect offspring development, however the weights of the dams in our study are consistent with those reported in other studies in which effects on offspring body weight, appetite, glucose-insulin homeostasis and blood pressure are reported [7,24] and we have previously shown in this model that pregnant females are hyperinsulinaemic and hypercholesterolaemic in late gestation [9]. We have previously suggested that one explanation might be maternal adaptations which occurred during pregnancy, so that although prior to conception, DIO dams were heavier and hyperglycaemic and hyperinsulinaemic when compared to control dams, they gained less weight and were no longer hyperglycaemic or hyperlipidaemic in late gestation [9,25]. Finally, although other studies report effects are present in F1 offspring by 6 months, it is possible that a phenotype might become more apparent in our model with ageing [26].

Sex differences in the response of males and females to diet-induced obesity are well-described in the literature [27] and sex specific effects in prenatal programming paradigms are also common [28-31]. The potential mechanisms have been recently reviewed [32] and may include sex differences in developmental trajectories and timing and the effects of sex steroids. Additionally, in our study, the effects of post-weaning exposure to an obesogenic diet were sex-specific. Whilst both sexes gained more weight on the cafeteria diet and had a substantial increase in fat pad weight compared to controls, there was a more severe metabolic phenotype in obese males, which showed profound hyperglycaemia and hyperinsulinaemia on glucose tolerance testing whilst females developed less severe hyperinsulinaemia and hyperglycaemia. Although it is well recognised that there are gender differences in cardiovascular disease risk associated with obesity in humans [33], most studies aimed at dissecting the mechanisms linking obesity and cardiovascular disease and at developing treatment strategies to ameliorate these consequences have been performed in males [33]. The processes accounting for these gender differences are not well defined but sex steroids presumably play a key, albeit complex role [34].

## Conclusions

In conclusion, we show that although post-weaning exposure to a cafeteria diet results in increased adiposity and metabolic derangement in both sexes, exposure to maternal overnutrition in early life has little effect to exacerbate this phenotype. Further studies designed to increase our understanding of what factors lead to the differences between models may help us understand the link between overnutrition in early life and later disease risk in humans and suggest ways to intervene to prevent the complications of exposure to overnutrition in early life.

## Competing interests

The authors declare that they have no competing interests.

## Authors’ contributions

VK, JEN, JRS and AJD contributed to study design. VK and AJD performed studies and data analysis. All authors contributed to writing and read and approved the final manuscript.
